# Signaling Cascade Involved in Rapid Stimulation of Cystic Fibrosis Transmembrane Conductance Regulator (CFTR) by Dexamethasone

**DOI:** 10.3390/ijms18081807

**Published:** 2017-08-19

**Authors:** Miriam Bossmann, Benjamin W. Ackermann, Ulrich H. Thome, Mandy Laube

**Affiliations:** Center for Pediatric Research Leipzig, Division of Neonatology, University of Leipzig, 04103 Leipzig, Germany; miriam.bossmann@medizin.uni-leipzig.de (M.B.); benjamin.ackermann@medizin.uni-leipzig.de (B.W.A.); ulrich.thome@medizin.uni-leipzig.de (U.H.T.)

**Keywords:** cystic fibrosis transmembrane conductance regulator, glucocorticoids, dexamethasone, airway, phosphoinositide 3-kinase, serum and glucocorticoid dependent kinase 1, protein kinase B, neural precursor cell expressed, developmentally downregulated 4-like

## Abstract

Impairment of mucociliary clearance with reduced airway fluid secretion leads to chronically inflamed airways. Cystic fibrosis transmembrane conductance regulator (CFTR) is crucially involved in airway fluid secretion and dexamethasone (dexa) has previously been shown to elevate CFTR activity in airway epithelial cells. However, the pathway by which dexa increases CFTR activity is largely unknown. We aimed to determine whether the increase of CFTR activity by dexa is achieved by non-genomic signaling and hypothesized that the phosphoinositide 3-kinase (PI3K) pathway is involved in CFTR stimulation. Primary rat airway epithelial cells and human bronchial submucosal gland-derived Calu-3 cells were analyzed in Ussing chambers and kinase activation was determined by Western blots. Results demonstrated a critical involvement of PI3K and protein kinase B (AKT) signaling in the dexa-induced increase of CFTR activity, while serum and glucocorticoid dependent kinase 1 (SGK1) activity was not essential. We further demonstrated a reduced neural precursor cell expressed, developmentally downregulated 4-like (NEDD4L) ubiquitin E3 ligase activity induced by dexa, possibly responsible for the elevated CFTR activity. Finally, increases of CFTR activity by dexa were demonstrated within 30 min accompanied by rapid activation of AKT. In conclusion, dexa induces a rapid stimulation of CFTR activity which depends on PI3K/AKT signaling in airway epithelial cells. Glucocorticoids might thus represent, in addition to their immunomodulatory actions, a therapeutic strategy to rapidly increase airway fluid secretion.

## 1. Introduction

Glucocorticoids (GCs) exert many physiological functions including regulation of pulmonary ion channels. The classical genomic mechanism of GC action is mediated by the cytosolic glucocorticoid receptor (GR). Different GR isoforms are produced by alternative splicing. In respiratory epithelial cells, human glucocorticoid receptor-α (hGR-α) is the predominant isoform, displaying steroid-binding activity. In contrast, hGR-β does not show ligand-binding activity [1] and inhibits the transcriptional activity of hGR-α by formation of transcription impairing hGR-α/hGR-β heterodimers [2]. Non-genomic effects of GC can also be mediated by proteins that dissociate from the cytosolic GR-multiprotein complex following GC binding [3]. In addition, rapid GC effects can be mediated by nonspecific interactions of GCs with cellular membranes and specific interactions with membrane-bound GRs [3].

Fluid homeostasis within the conducting segments of the airways is achieved by controlled lung fluid production and absorption, mediated by ion channels like the cystic fibrosis transmembrane conductance regulator (CFTR). Disturbances can affect mucociliary clearance (MCC), which is based on the correct height and thickness of the periciliary liquid layer [4,5]. In the case of dysfunctional CFTR, MCC is impaired, leading to chronically inflamed airways [6]. CFTR dysfunctions may develop due to environmental toxins, cigarette smoke, genetic diseases like cystic fibrosis or acquired pathologies like chronic obstructive pulmonary disease (COPD) [7,8]. Therefore, determining GC effects on the secretion process in the upper airways in relation to MCC is important since anti-inflammatory therapies with GCs are common for chronic airway inflammatory conditions, such as infections and allergies.

CFTR is a plasma membrane cyclic AMP (cAMP)-regulated chloride (Cl^−^) channel located at the apical membrane of epithelial cells in several tissues. To date, little is known about the effect of hormones on CFTR function, which appears to differ between the distal and the proximal airways. Our previous study showed that GCs reduced CFTR expression and activity in distal lung epithelial cells, which are responsible for gas exchange and fluid absorption [9]. In contrast, GCs stimulated CFTR activity in the air conducting proximal airways, which are responsible for MCC. Although GCs increased CFTR activity, its gene expression was also reduced, yet not as pronounced as in the distal epithelia. These results led us to address the pathway that results in an increased CFTR activity in bronchial/tracheal epithelia. Since GCs negatively regulated CFTR gene expression, non-genomic pathways were assumed. In agreement, GCs have been shown to induce effects within minutes, incompatible with effects on gene transcription by GRs [10,11,12]. Supporting our hypothesis, studies showed that GCs increased CFTR protein expression two-fold, which was attributed to an altered chaperone interaction resulting in increased CFTR protein trafficking [13]. Moreover, in the transformed cystic fibrosis (CF) bronchial epithelial cell line CFBE41o-, the GC-induced increase of serum and GC dependent kinase 1 (SGK1) protein abundance enhanced ΔF508-CFTR [14] and wildtype (wt) CFTR membrane expression by inhibiting their endocytic retrieval [15]. SGK1 phosphorylates and thereby inhibits the ubiquitin ligase neural precursor cell expressed, developmentally down-regulated protein 4-like (NEDD4L), a mechanism possibly responsible for the elevated CFTR protein abundance. These studies in combination with our previous results suggest that kinase signaling is majorly involved in stimulated CFTR activity induced by GCs in airway epithelial cells. We thus aimed to determine signaling pathways leading to stimulation of CFTR activity by GCs using dexamethasone (dexa) in primary rat airway epithelial cells and human bronchial epithelial Calu-3 cells.

## 2. Results

### 2.1. The Phosphoinositide 3-Kinase (PI3K) Pathway Is Involved in the Dexa-Stimulated Cystic Fibrosis Transmembrane Conductance Regulator Activity

**Figure 1:**
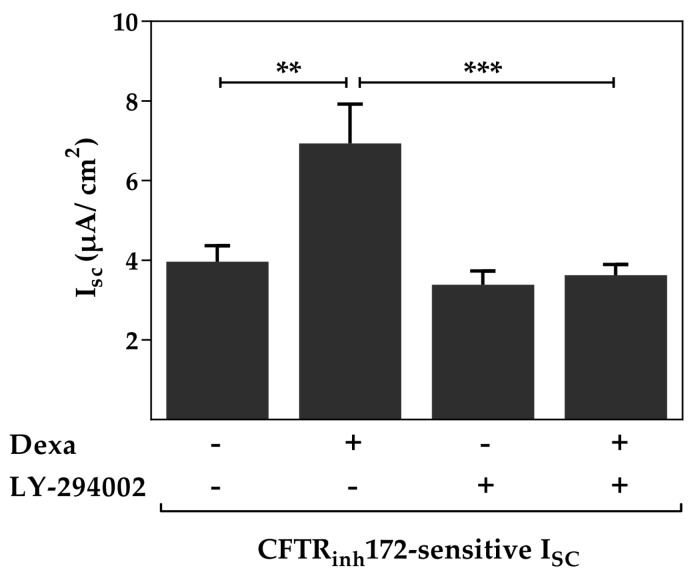
Phosphoinositide 3-kinase (PI3K) contributes to the increased cystic fibrosis transmembrane conductance regulator (CFTR) activity induced by dexa. Primary airway epithelial cells were treated with 100 nM dexa and LY-294002 (10 µM) for 24 h. Data bars represent mean + standard error of the mean (SEM) of I_SC_. Dexa increased the CFTR_inh_172-sensitive I_SC_. Addition of LY-294002 prevented the dexa-induced increase of CFTR activity (*n* = 20–25, ** *p* < 0.01, *** *p* < 0.001, analysis of variance (ANOVA) with Tukey’s *post hoc* test).

To determine the signaling pathway of dexa action we used different kinase inhibitors. First, dexa increased CFTR activity in Ussing chamber measurements, as demonstrated by the significantly elevated CFTR_inh_172-sensitive I_SC_, in primary airway epithelial cells (*p* < 0.01, Figure 1). LY-294002 was used to block the phosphoinositide 3-kinase (PI3K) and measurements showed that dexa was unable to increase CFTR activity when LY-294002 was present (*p* < 0.001). These experiments showed that the PI3K activity is indispensable for the stimulating effect of dexa on CFTR activity.

### 2.2. Serum and Glucocorticoid Dependent Kinase 1 Is Not Involved in the Dexa-Stimulated CFTR Activity

**Figure 2:**
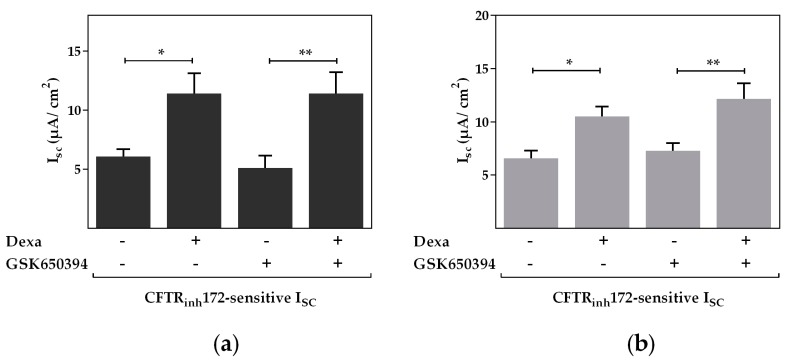
Serum and glucocorticoid dependent kinase 1 activity is not involved in the dexa-stimulated CFTR activity. Cells were treated with 100 nM dexa and GSK650394 (10 µM) for 24 h. Data bars represent mean + SEM of I_SC_. (**a**) In primary airway epithelial cells, the CFTR_inh_172-sensitive I_SC_ was increased by dexa. Addition of GSK650394 displayed no effect on the dexa-induced increase of CFTR activity (*n* = 10–15, * *p* < 0.05, ** *p* < 0.01, ANOVA with Tukey’s *post hoc* test); (**b**) in Calu-3 cells CFTR_inh_172-sensitive I_SC_ was increased by dexa. In agreement, GSK650394 displayed no effect on the dexa-induced increase of CFTR activity (*n* = 24–31, * *p* < 0.05, ** *p* < 0.01, ANOVA with Tukey’s *post hoc* test).

In contrast to the contribution of PI3K, the inhibitor of SGK1, GSK650394 had no effect on the dexa-stimulated CFTR activity in either primary airway epithelial or Calu-3 cells. Dexa was still able to significantly increase CFTR activity even in the presence of GSK650394 (*p* < 0.05, *p* < 0.01, Figure 2a,b).

**Figure 3:**
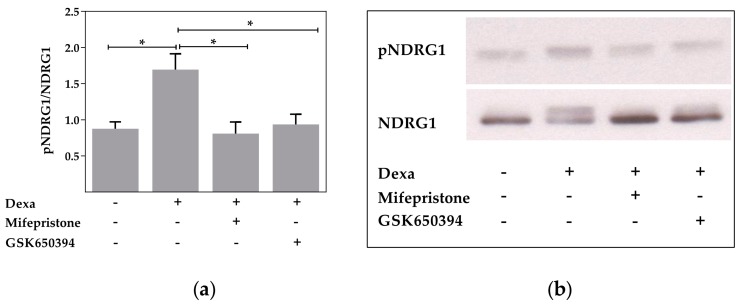
SGK1 activity is elevated by dexa and reduced by GSK650394. (**a**) Normalized densitometric evaluation of n-myc downregulated gene 1 (NDRG1) and pNDRG1 Western blots. Calu-3 cells were treated with 100 nM dexa and GSK650394 (10 µM) or mifepristone (10 µM) for 24 h (*n* = 4; * *p* < 0.05 by *t*-test); (**b**) western blot of pNDRG1 and total NDRG1 resulted in bands of 46 and 48 kDa.

It is known that SGK1 activity is elevated by GC exposure. Western blot measurements showed that dexa significantly increased phosphorylation of n-myc downregulated gene 1 (NDRG1), a specific substrate of SGK1, and thus representing an elevated SGK1 enzyme activity compared with control cells (*p* < 0.05, Figure 3a). Therefore, the results do support an activation of SGK1 by dexa. However, both the addition of mifepristone to inhibit the GR and GSK650394 suppressed the increased SGK1 activity induced by dexa, as shown by a significant reduction of phosphorylated NDRG1, which reached unstimulated control levels (*p* < 0.05, Figure 3a,b). The Western blot measurement therefore shows that dexa increases SGK1 activity and that GSK650394 is effective in blocking SGK1 activation by dexa. Since the effect of dexa in Ussing chamber measurements persisted after the application of GSK650394, the activity of SGK1 is not decisively involved in dexa-stimulated CFTR activity.

### 2.3. Protein Kinase B Activity Is Involved in the Dexa-Stimulated CFTR Activity

**Figure 4:**
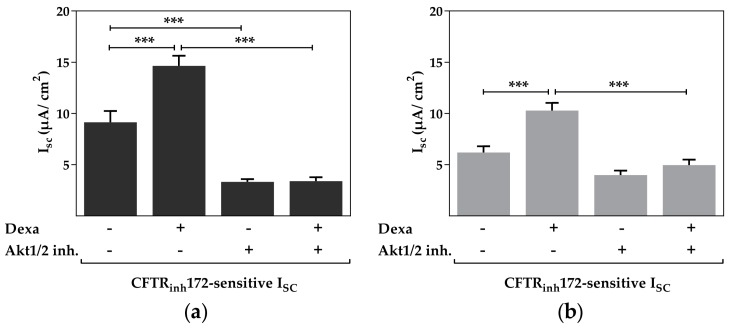
Akt1/2 kinase inhibitor prevents the increase of CFTR activity induced by dexa. Cells were treated with 100 nM dexa and Akt1/2 kinase inhibitor (10 µM) for 24 h. Data bars represent the mean + SEM of I_SC_. (**a**) In primary airway epithelial cells, the CFTR_inh_172-sensitive I_SC_ was increased by dexa. Addition of Akt1/2 kinase inhibitor prevented the dexa-induced increase of CFTR activity (*n* = 11–14; *** *p* < 0.001, ANOVA with Tukey’s *post hoc* test); (**b**) in Calu-3 cells the CFTR_inh_172-sensitive I_SC_ was increased by dexa. Likewise, addition of Akt1/2 kinase inhibitor prevented the dexa-induced increase of CFTR activity (*n* = 36–37, *** *p* < 0.001, ANOVA with Tukey’s *post hoc* test).

PI3K signaling affects ion channel activity through SGK1 and/or protein kinase B (AKT). We thus checked for an involvement of AKT in the dexa-stimulated CFTR activity. Akt1/2 kinase inhibitor was used to block AKT activation which decreased CFTR activity in control and dexa-stimulated cells. Similar results were obtained in primary airway epithelial and Calu-3 cells (*p* < 0.05, *p* < 0.001, Figure 4a,b). Furthermore, dexa was unable to increase CFTR activity when Akt1/2 kinase inhibitor was present. Therefore, in addition to PI3K, AKT is indispensable for enhancement of CFTR activity by dexa.

**Figure 5:**
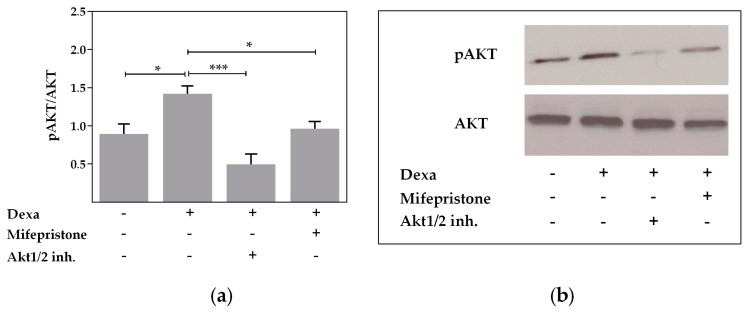
AKT activity is elevated by dexa. (**a**) Normalized densitometric evaluation of pAKT and AKT Western blots. Calu-3 cells were treated with 100 nM dexa and Akt1/2 kinase inhibitor (10 µM) or mifepristone (10 µM) for 24 h. Addition of mifepristone and Akt1/2 kinase inhibitor blocked the increased AKT activity induced by dexa (*n* = 4; * *p* < 0.05; *** *p* < 0.001 by *t*-test); (**b**) western blot of pAKT and total AKT resulted in bands of 60 kDa.

To verify an involvement of AKT in the stimulation by dexa, we analyzed the phosphorylation of AKT with Western blot. Western blot measurements showed that dexa significantly increased phosphorylation of AKT at Ser473, representing an elevated AKT enzyme activity (*p* < 0.05, Figure 5a,b). Both the addition of mifepristone and Akt1/2 kinase inhibitor blocked the increased AKT activity induced by dexa, as shown by a significant reduction of phosphorylated AKT, which reached unstimulated control levels (*p* < 0.05, *p* < 0.001, Figure 5a,b). The Western blot measurement therefore shows that dexa increases AKT activity and that both, Akt1/2 kinase inhibitor and GR inhibition are effective in blocking AKT activation by dexa.

### 2.4. Neural Precursor Cell Expressed, Developmentally Downregulated 4-Like Activity Is Reduced by Dexa

**Figure 6:**
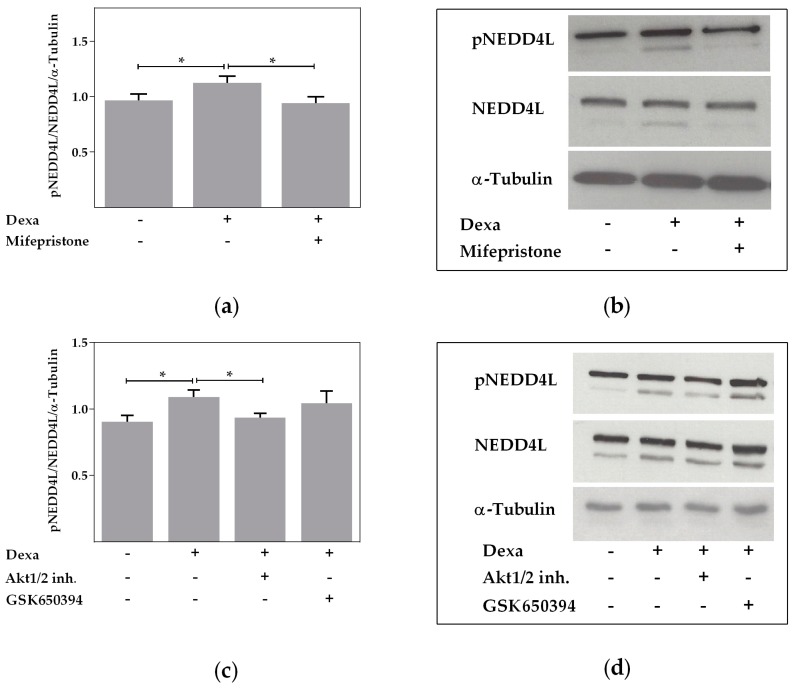
Dexa increases phosphorylation of neural precursor cell expressed, developmentally downregulated 4-like (NEDD4L). (**a**) Normalized densitometric evaluation of pNEDD4L, NEDD4L and α-tubulin Western blots. Calu-3 cells were treated with 100 nM dexa and mifepristone (10 µM) for 24 h (*n* = 5; * *p* < 0.05 by *t*-test); (**b**) western blot of pNEDD4L and total NEDD4L resulted in bands of 110 and 135 kDa. Western blot of α-tubulin resulted in bands of 52 kDa; (**c**) normalized densitometric evaluation of pNEDD4L, NEDD4L and α-tubulin Western blots. Calu-3 cells were treated with 100 nM dexa and Akt1/2 kinase inhibitor (10 µM) or GSK650394 (10 µM) for 24 h (*n* = 5; * *p* < 0.05 by *t*-test); (**d**) western blot of pNEDD4L and total NEDD4L resulted in bands of 110 and 135 kDa. Western blot of α-tubulin resulted in bands of 52 kDa.

NEDD4L degrades membrane-bound ion channels and thereby decreases channel activity. NEDD4L activity can be affected by phosphorylation through AKT or SGK1 that reduces its enzyme activity. We thus analyzed if phosphorylation of NEDD4L is affected by dexa in Calu-3 cells and found phosphorylation of NEDD4L to be significantly increased by dexa (*p* < 0.05, Figure 6a–d). Mifepristone prevented the dexa-stimulated increase of phosphorylated NEDD4L (*p* < 0.05, Figure 6a,b). Furthermore, AKT inhibition by Akt1/2 kinase inhibitor reduced phosphorylation of NEDD4L in dexa-stimulated cells, which reached unstimulated control levels (*p* < 0.05, Figure 6c,d). In agreement with our previous results, SGK1 inhibition by GSK650394 had no significant effect on phosphorylated NEDD4L in dexa-stimulated cells (Figure 6c,d). In conclusion, the Western blots show that dexa decreases NEDD4L activity by increasing its phosphorylation, which is blocked by GR and AKT inhibition.

### 2.5. Rapid Effects of Dexa on CFTR Activity

**Figure 7:**
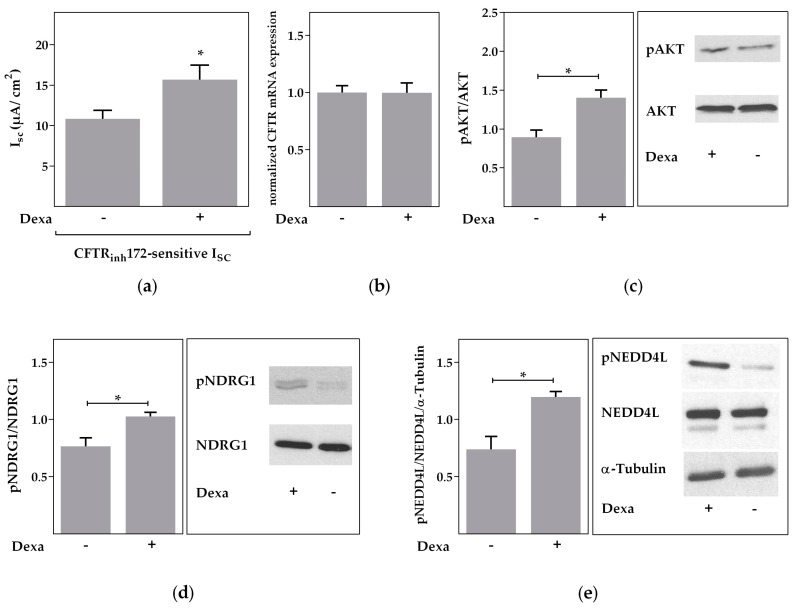
Rapid effects of dexa on CFTR activity. Calu-3 cells were treated with 100 nM dexa for 30 min. Data bars represent the mean + SEM. (**a**) The CFTR_inh_172-sensitive I_SC_ was significantly increased by dexa (*n* = 60, * *p* < 0.05 by t-test with Welch’s correction); (**b**) CFTR mRNA expression was not affected by dexa (*n* = 6); (**c**) normalized densitometric evaluation of pAKT and AKT Western blots (*n* = 3; * *p* < 0.05 by *t*-test). Western blot of pAKT and total AKT resulted in bands of 60 kDa; (**d**) normalized densitometric evaluation of pNDRG1 and NDRG1 Western blots (*n* = 3; * *p* < 0.05 by *t*-test). Western blot of pNDRG1 and total NDRG1 resulted in bands of 46 and 48 kDa; (**e**) normalized densitometric evaluation of pNEDD4L, NEDD4L and α-tubulin Western blots (*n* = 3; * *p* < 0.05 by *t*-test). Western blot of pNEDD4L and total NEDD4L resulted in bands of 110 and 135 kDa. Western blot of α-tubulin resulted in bands of 52 kDa.

The described signaling pathway of dexa should be able to render its effect within very short time frames. To test this hypothesis, we stimulated Calu-3 cells with dexa for only 30 min instead of 24 h. Even within this short time frame, CFTR activity was significantly increased by dexa as demonstrated in Ussing chamber measurements (*p* < 0.05, Figure 7a). On the other hand, CFTR mRNA expression was unaffected after short term exposure to dexa (Figure 7b). Finally, phosphorylation of AKT, NDRG1 and NEDD4L was demonstrated within 30 min of dexa application (*p* < 0.05, Figure 7c–e), further supporting a rapid kinase-dependent signaling pathway of dexa stimulation.

## 3. Discussion

CFTR channels are critical for airway function and GCs have been shown to stimulate CFTR activity in airway epithelial cells [9]. This is especially true for chloride secretion, and therefore maintenance of airway surface liquid layer is controlled by CFTR. Regarding the broad clinical use of GCs for several airway disorders, it is of central importance to elucidate their impact on epithelial physiology. Herein, we aimed to determine whether the increase of CFTR activity by dexa is achieved by non-genomic signaling and hypothesized that the PI3K pathway is involved in CFTR stimulation. We demonstrated that PI3K and AKT are indispensable for the stimulatory action of GCs, while SGK1 is not majorly involved in the signaling cascade leading to enhanced CFTR activity. We further showed that GCs increased phosphorylation of AKT, SGK1 and NEDD4L in airway epithelial cells, representing an enhanced activity of AKT and SGK1 and a decreased NEDD4L protein function. AKT inhibition prevented the increased phosphorylation of NEDD4L, while SGK1 inhibition was unable to prevent NEDD4L phosphorylation. Finally, stimulation of CFTR activity by GCs was demonstrated within 30 min, further supporting rapid signaling by kinases.

Our previous study demonstrated that GCs exhibit different effects on proximal and distal airway compartments. Within the proximal air-conducting compartment, GCs promote fluid secretion by enhancing CFTR activity as shown in Calu-3 and primary airway epithelial cells [9]. Airway fluid secretion enhances MCC by maintaining the airway surface liquid layer [4,5]. In accordance with our results, it was shown that dexa increased CFTR protein expression two-fold which was attributed to an altered chaperone interaction resulting in increased CFTR protein trafficking, while CFTR gene expression was also reduced [13]. In distal airways, the absorptive phenotype predominates enabling gas exchange in the alveoli. It might thus make physiological sense that GCs reduced alveolar CFTR activity in distal lung cells, whereas alveolar ENaC activity was increased [16], promoting fluid absorption to ensure efficient gas exchange. Since GCs reduced CFTR gene expression in distal, but also in proximal lung compartments, the stimulation of CFTR activity in airway epithelial cells is most likely mediated by non-genomic mechanisms.

Our results showed that PI3K activity is essential for the stimulatory effect of GCs on CFTR activity in primary airway epithelial cells. Activation of receptor tyrosine kinases results in activation of PI3K. The catalytic domain of PI3K subsequently converts phosphatidylinositol (3,4)-biphosphate (PIP_2_) to phosphatidylinositol (3,4,5)-triphosphate (PIP_3_), leading to activation of phosphoinositide-dependent protein kinase (PDK1) and mammalian target of rapamycin complex 2 (mTORC2), followed by phosphorylation and activation of AKT. Phosphorylation of AKT at Ser473 by mTORC2 facilitates phosphorylation of Thr308 by PDK1 [17]. Inhibition of the PI3K by LY294002 [18], prevented the GC-mediated increase of CFTR activity. Basal CFTR activity was not affected by LY294002 in primary airway epithelial cells, suggesting that PI3K activity is not essential for basal CFTR function in these cells. These results are in contrast to Calu-3 cells in which both the GC-induced and the basal CFTR activity were affected by PI3K inhibition [9]. Differences between human submucosal gland-derived Calu-3 cells and rat primary airway cells might be due to additional mechanisms maintaining basal CFTR activity. Confirming our findings, LY294002 was shown to inhibit the forskolin-induced phosphorylation of CFTR in duodenal epithelial cells as well as CFTR trafficking to the membrane [18]. Further kinase involvement, like that of PIKfyve [19] and CK2 [20] or participation of P2Y2 receptors [21,22] might contribute to maintaining basal CFTR activity, even after PI3K inhibition, in primary airway epithelial cells.

Downstream of PI3K, involvement of SGK1 and AKT in regulation of epithelial ion channels has been reported. SGK1 is a serin/threonine kinase controlled by several stimuli including GCs. SGK1 plays an important role in regulating ion transport as well as cell metabolism and tumor growth, and is activated via PI3K, PDK1 and mTORC2 [23,24,25,26]. Nevertheless, Ussing chamber measurements demonstrated that SGK1 inhibition had no effect on CFTR stimulation by GCs in primary airway epithelial and Calu-3 cells, since dexa-stimulated CFTR currents were still elevated even after SGK1 inhibition. Furthermore, basal CFTR activity was not affected by SGK1 inhibition. Western blot analysis confirmed that SGK1 was indeed effectively inhibited by GSK650394, preventing the increased phosphorylation of NDRG1 induced by GCs. In addition, GR inhibition by mifepristone also prevented the increased SGK1 activity induced by GCs. This indicates that SGK1 is not involved in CFTR regulation. In contrast, other studies demonstrated that SGK1 enhances the functional activity of CFTR when coexpressed in *Xenopus* oocytes [26,27]. In pancreatic cells, dexa was shown to elevate the functional expression of wt- and ΔF508-CFTR by increasing total protein expression, cell surface expression and channel half-life [28]. This study showed that inhibition of either the GR or the PI3K and knock-down of SGK1 blocks the effect of dexa on CFTR trafficking [28]. In agreement with this, the GC-induced increase of SGK1 protein abundance enhanced ΔF508-CFTR [14] and wt-CFTR membrane expression in CFBE41o- cells by inhibiting their endocytic retrieval [15]. Herein, we did not observe a major contribution of SGK1 to the enhanced CFTR activity, although we also did not determine CFTR trafficking or membrane abundance. Therefore, CFTR activity and protein abundance might not be directly comparable. Furthermore, it is known that SGK1 contributes to the stimulating effect of insulin on Na^+^ transport. However, in a previous study no major contribution of SGK1 to the stimulating effect of insulin on Na^+^ transport was observed in primary alveolar cells [29]. These results were supported by H441 cells, which are of respiratory origin, while in renal mpkCCD cells the same study showed that the impact of insulin was critically dependent on activation of SGK1 [30]. It was therefore suggested that SGK1 exhibits cell type-specific effects [30] or dependence on the presence of local anchoring proteins [31], which might also explain the differing results obtained in our study.

In contrast to SGK1, AKT, another kinase downstream of PI3K, was indispensable for the increased CFTR activity as shown in Ussing chamber measurements. AKT inhibition by Akt1/2 kinase inhibitor markedly decreased CFTR activity in both dexa-stimulated and unstimulated control cells. These results demonstrate that CFTR activity is largely dependent on AKT activity. Furthermore, dexa is unable to stimulate CFTR activity in the presence of Akt1/2 kinase inhibitor. Western blot analysis showed increased Ser473-phosphorylation and thus activation of AKT after incubation with GCs, which was prevented by Akt1/2 kinase inhibitor. Inhibition of the GR by mifepristone reduced AKT phosphorylation accordingly. Akt1/2 kinase inhibitor is a highly selective noncompetitive inhibitor of AKT [32], which prevents the conformational change, triggered by binding of PIP_3_ to the pleckstrin homology domain of AKT isoforms, which allows PDK1 and mTORC2 to phosphorylate and activate AKT [33]. However, it has been reported that Akt1/2 kinase inhibitor might also inhibit SGK1, since it was shown to prevent phosphorylation of NDRG1 [34], even though others reported no inhibition of SGK1 with Akt1/2 kinase inhibitor at concentrations as high as 250 µM [32]. In our experiments, we assume that a possible unspecific effect of Akt1/2 kinase inhibitor is negligible, since direct inhibition of SGK1 by GSK650394 did not affect CFTR activity. Therefore, the complete suppression of the GC effect by Akt1/2 kinase inhibitor was attributable to the inhibition of AKT.

NEDD4L ubiquitinates membrane proteins, including ion channels, leading to increased internalization and degradation of its target proteins [35,36,37]. Western blot analysis demonstrated an increased phosphorylation of NEDD4L induced by dexa in Calu-3 cells. Phosphorylation of NEDD4L at Ser468 increases its binding affinity to the regulatory protein 14-3-3, which in turn suppresses ubiquitin E3 ligase activities of NEDD4L by inhibiting formation of the enzyme/substrate complex [38]. In agreement to Ussing chamber measurements, GR inhibition prevented the effect of dexa on NEDD4L phosphorylation. Studies showed that Na^+^ transport is strongly affected by NEDD4L and phosphorylation of NEDD4L thus increases epithelial Na^+^ channel (ENaC) membrane abundance by preventing its endocytic retrieval [31,39]. Regarding the relationship between NEDD4L and CFTR, different results were published. In pancreatic CFPAC-1 cells, expression of ΔF508-CFTR was increased by knockdown of NEDD4L [28]. In contrast, neither overexpression nor silencing of NEDD4L affected wt-CFTR apical membrane abundance or Cl^−^ currents in *Xenopus* oocytes or CFBE-wt cells [40]. However, the authors also suggested that NEDD4L might regulate CFTR indirectly by ubiquitinating positive or negative regulators of CFTR membrane density or activity [40]. More precisely, a small but significant increase in CFTR membrane abundance without a concomitant increase in CFTR Cl^−^ currents was noted upon NEDD4L overexpression, while CFTR currents increased without elevations in CFTR membrane density with NEDD4L knockdown. The authors suggested that overexpression of NEDD4L might result in increased ubiquitination and degradation of negative regulators of CFTR abundance as well as positive regulators of CFTR activity, resulting in no net increase in CFTR currents. In accordance, NEDD4L knockdown might reduce ubiquitination and degradation of positive regulators of CFTR activity, enhancing CFTR currents [40]. The proposed suggestion that CFTR protein abundance and channel function might not be directly comparable possibly explains the different results obtained in previously published studies as well as the unaffected CFTR activity by SGK1 inhibition as described above.

Furthermore, AKT and SGK1 are both reported to inhibit the actions of NEDD4L by phosphorylation [31,39,41]. Inhibition of AKT by Akt1/2 kinase inhibitor prevented the elevated NEDD4L phosphorylation induced by dexa, supporting a major involvement of AKT in GC actions. On the other hand, inhibition of SGK1 by GSK650394 had no major effect on NEDD4L phosphorylation, further questioning a contribution of SGK1 in dexa stimulation. Our results therefore support a PI3K/AKT dependent stimulation of CFTR activity by dexa, which is possibly mediated by inhibition of NEDD4L. A direct interaction between CFTR and NEDD4L or NEDD4L and AKT was not determined in this study and must be addressed in future studies. Finally, CFTR activity was significantly increased by dexa within 30 min as demonstrated in Ussing chamber measurements. Interestingly, activation of AKT has been demonstrated within 2 min after GC stimulation in alveolar A-549 cells, which was prevented by PI3K inhibition [10]. We also showed an increased phosphorylation of AKT, NEDD4L and NDRG1 by dexa within 30 min, whereas CFTR mRNA expression was not affected. These results further support the proposed kinase signaling pathway.

Non-genomic effects are generally believed to take place within 30 min of GC application due to the considerable latency of genomic steroid effects [42]. We did not analyze which GR isoform is responsible for the observed effects, but it is important to note that increased hGR-β expression has been correlated with several diseases related to GC resistance [43]. Future studies thus need to characterize the involved GR isoforms and determine whether a short-term GC exposure yielding to rapid increases of CFTR activity harbors a risk for GC-resistant lung disease.

## 4. Materials and Methods

### 4.1. Tissue Preparation

All animal care and experimental procedures were approved by the institutional review board (Landesdirektion Leipzig, Permit Number: T36/13, 21th Dec 2012). Sprague-Dawley rats were bred at the Medical Experimental Center (MEZ) of Leipzig University. Animals were housed in rooms with a controlled temperature (22 °C), humidity (55%) and 12 h light-dark cycle. Food and water were freely available. Rats were euthanized by carbon dioxide inhalation.

### 4.2. Isolation of Primary Airway Epithelial Cells

Isolation of primary airway epithelial cells has been described previously [44]. Briefly, the trachea proximal to the bronchial bifurcation was isolated from euthanized male Sprague-Dawley rats (140–200 g). Esophageal remnants and adherent adipose tissue were removed. The trachea was opened longitudinally and rinsed with Dulbecco's Modified Eagle's Medium (DMEM) (Thermo Fisher Scientific, Darmstadt, Germany) before incubation in DMEM with 0.1% protease XIV (# P5147, Sigma-Aldrich, Munich, Germany), 0.01% DNase I (# D5025, Sigma-Aldrich) and antibiotic-antimycotic (Thermo Fisher Scientific), a mixture of penicillin (100 U/mL), streptomycin (100 µg/mL) and amphotericin B (0.25 µg/mL), for 21 h at 4 °C while shaking. The digestion was stopped by the addition of 1 Vol. fetal bovine serum (FBS) (Biochrom, Berlin, Germany). The trachea was then agitated and scraped with a cell scraper to detach the airway epithelial cells. The obtained cell suspension was centrifuged twice at 500× *g* for 5 min and re-suspended in cell culture media. Cell culture media consisted of DMEM-F12 (Thermo Fisher Scientific) with 1 µg/mL insulin (# 91077C, Sigma-Aldrich, Taufkirchen, Germany), 7.5 µg/mL transferrin (# 354204, Corning GmbH, Wiesbaden, Germany), 1 µM hydrocortisone (# H0888, Sigma-Aldrich), 30 nM 3,5,3′-triiodothyronine (# T6397, Sigma-Aldrich), 25 ng/mL epidermal growth factor (# 354052, Corning GmbH), 10 ng/mL endothelial cell growth supplement (# 354006, Corning GmbH), and antibiotic-antimycotic, which was supplemented (1:1) with 3T3 fibroblast (from ATCC^®^, Manassas, VA, USA, # CCL-92)-conditioned DMEM containing 2% FBS. Cells were seeded on collagen-coated (human placental collagen type IV, # C5533, Sigma-Aldrich) permeable supports at a density of 2 × 10^5^ cells per Snapwell insert (Costar, # 3407, surface area 1.1 cm^2^, Corning GmbH). Medium was changed after the first 24 h and then every two days. Measurements were done approximately 10 days after plating, when the transepithelial resistance (*R*_te_) reached values >300 Ω·cm^2^. *R*_te_ was measured during medium change with an EVOM epithelial voltohmmeter (World Precision Instruments, Sarasota, FL, USA) with STX-2 chopstick electrodes. Cells subjected to different experimental conditions were always age matched, treated equally and recorded simultaneously.

### 4.3. Culture of Cell Lines

Calu-3 cells (from ATCC, # HTB-55), derived from human bronchial submucosal glands, were kindly provided by Getu Abraham (Institute of Pharmacology, Pharmacy and Toxicology, Faculty of Veterinary Medicine, University of Leipzig). Calu-3 cells (passage 23–30) were cultured in DMEM-F12 with 10% FBS, penicillin (100 U/mL), streptomycin (100 µg/mL) and 1% non-essential amino acids (Thermo Fisher Scientific), and passaged 1–2 times weekly. Calu-3 cells were seeded at a density of 5 × 10^5^ cells per Snapwell insert and 1 × 10^6^ cells per Transwell insert (Costar, # 3412, surface area 4.6 cm^2^). After 10 days, Calu-3 cells were subjected to air-liquid interface conditions and *R*_te_ was measured every two days during medium change. Measurements and protein isolation were done approximately 14–21 days after plating of Calu-3 cells on permeable supports, when *R*_te_ reached values >300 Ω·cm^2^.

For all analyzed cell types serum-free complete medium (Cellgro, Corning GmbH), supplemented with dexa (100 nM, # D4902, Sigma-Aldrich) was added 24 h before measurement or as stated otherwise. To determine the involvement of the PI3K, the inhibitor LY-294002 (10 µM, # 1130 TOCRIS Bioscience, Bristol, UK) was used. Furthermore, Akt1/2 kinase inhibitor (10 µM, # A6730, Sigma-Aldrich) was used to inhibit the AKT, GSK650394 (10 µM, # 3572, TOCRIS Bioscience) to inhibit the SGK1 and mifepristone (10 µM, # M8046, Sigma-Aldrich) to inhibit the GR.

### 4.4. Electrophysiological Measurements

A detailed description of Ussing chamber measurement procedures is reported elsewhere [45]. Experiments were included in the data analyses only when *R*_te_ exceeded 300 Ω·cm^2^ throughout the measurement. Ussing chambers were filled with a ringer solution containing: Na^+^ 145 mM, K^+^ 5 mM, Ca^2+^ 1.2 mM, Mg^2+^ 1.2 mM, Cl^−^ 125 mM, HCO_3_^−^ 25 mM, H_2_PO_4_^−^ 3.3 mM, HPO_4_^2−^ 0.8 mM (pH 7.4). The basolateral side contained 10 mM glucose whereas 10 mM mannitol was used in the apical compartment. Equivalent short-circuit currents (I_SC_) were assessed every 20 s by measuring transepithelial voltage (*V*_te_) and *R*_te_ using a transepithelial current clamp (Physiologic Instruments, San Diego, CA, USA), and calculating the quotient I_SC_ = *V*_te_/*R*_te_. Amiloride (10 µM, # A-7410, Sigma-Aldrich), an inhibitor of ENaC, was added to the apical compartment to inhibit amiloride-sensitive Na^+^ channels. Forskolin (10 µM, # F-6886, Sigma-Aldrich) was added to the apical compartment to increase the intracellular cyclic adenosine monophosphate (cAMP) concentration and thereby activate cAMP-sensitive ion channels like CFTR. Finally, CFTR_inh_172 (10 µM, # 3430, TOCRIS Bioscience) was applied apically to determine the CFTR_inh_172-sensitive I_SC_, a measure of CFTR activity. Amiloride was dissolved in water; forskolin, CFTR_inh_172, mifepristone, GSK650394, Akt1/2 kinase inhibitor and LY-294002 were prepared in dimethyl sulfoxide (DMSO) and dexamethasone in 100% ethanol. Control monolayers were treated with the respective solvent to exclude solvent influence on the evoked responses.

### 4.5. Western Blot Analyses

Calu-3 cell Transwell inserts were placed on ice, washed with ice-cold phosphate buffered saline solution and overlaid with 250 µL of lysis buffer containing 50 mM Tris (pH 7.4), 150 mM NaCl, 1 mM ethylenediaminetetraacetic acid (EDTA), 1% NP-40 substitute, 0.25% sodium deoxycholate, 1 mM Na_3_VO_4_, 1 mM NaF and the protease inhibitor cocktail Roche complete (Roche GmbH, Mannheim, Germany). After 30 min, filters were excised from their holders and transferred into Eppendorf tubes, carefully collecting all fluid that had flown off. The tubes were freeze-thawed three times, and then centrifuged at 12,000× *g* and 4 °C for 10 min to accumulate the fluid at the bottom of the tubes. The almost dry filters were discarded, leaving the protein-rich lysate. Protein content was measured against bovine serum albumin standards in triplicate by a standard protein assay (BCA, Pierce, Rockford, IL, USA) on 96-well plates with an automatic plate reader. Proteins were separated by sodium dodecyl sulfate polyacrylamide gel electrophoresis (SDS-PAGE) through an 8 or 10% gel (40 µg protein per lane) and transferred onto nitrocellulose membrane. Blots were incubated overnight at 4 °C with the following primary antibodies, diluted according to the manufacturer’s standard protocol. Phosphorylation of NDRG1 was used to determine SGK1 enzyme activity [30,46,47] and detected with phospho-NDRG1 antibody (# 3217, Cell Signaling Technology, Inc., Danvers, MA, USA), when phosphorylated at Thr346, and total NDRG1 antibody (# 5196, Cell Signaling Technology, Inc.). Phosphorylation of AKT was analyzed using antibodies against phospho-AKT at Ser473 (# 9271, Cell Signaling Technology, Inc.), and AKT (# 9272, Cell Signaling Technology, Inc., both kindly provided by A. Garten). Phosphorylation of NEDD4L was measured with antibodies against phospho-NEDD4L at Ser448 (# 8063, Cell Signaling Technology, Inc.), total NEDD4L (# 4013, Cell Signaling Technology, Inc.) and α-tubulin (# 2144, Cell Signaling Technology, Inc.). Suitable secondary antibodies conjugated to horseradish peroxidase (HRP) were used to detect primary antibodies. HRP activity was analyzed by enhanced chemiluminescence (ECL, Amersham, Piscataway, NJ, USA) on x-ray film and band intensity was measured by densitometry using Image-J (National Institutes of Health (NIH), Bethesda, MD, USA).

### 4.6. Measurement of CFTR mRNA Expression

Total RNA was isolated using the PureLink RNA Mini Kit (Thermo Fisher Scientific) and treated with DNase I (Thermo Fisher Scientific) according to the manufacturer’s instructions. Reverse transcription was carried out employing the Maxima H Minus DNA Synthesis Kit (ThermoFisher Scientific). The SYBR Select Master Mix (ThermoFisher Scientific) was used for qRT-PCR, following the manufacturer’s instructions. Reactions were conducted with the IQ5 rtPCR Detection System (BioRad, Munich, Germany). Transcripts of target genes were amplified using the gene-specific primers for human CFTR 5′-GGGCTGTGTCCTAAGCCATGGCCA-3′ and 5′-GATGGCTTGCCGGAAGAGGCTCC-3′. Absolute quantification was performed using a several fold dilution of target specific plasmid-DNA as internal standard curve. The resulting molecule concentrations were normalized to a reference gene (Mrps18a: mitochondrial ribosomal protein S18a). Constant expression of Mrps18a was confirmed against other common reference genes. The fold change of mRNA levels was calculated with the relative standard curve method. Measurements were performed in technical triplicates and six biological replicates. Melting curves and gel electrophoresis of the PCR products were routinely performed to determine the specifity of the PCR reaction.

### 4.7. Statistical Analyses

For statistical analyses, GraphPad Prism (version 5.03; GraphPad Software, Inc., San Diego, CA, USA) was used. Differences among groups treated with dexamethasone and controls were evaluated by unpaired *t*-test, or analysis of variance (ANOVA) followed by Tukey’s *post hoc* test, as appropriate. A probability of *p* < 0.05 was considered significant for all statistical analyses.

## 5. Conclusions

The presented results support a rapid, most likely non-genomic signaling pathway of dexa stimulation via PI3K/AKT in airway epithelial cells. Rapid CFTR regulation by GCs might alter the relevance of GCs in acute treatment of airway disorders. Currently, GCs are widely used to treat inflammatory exacerbations, but the fast response demonstrated in this study possibly indicates an additional rapid effect on MCC. Improving disturbed MCC by enhanced CFTR activity and Cl^−^ secretion resulting in elevated fluid secretion might aid clearance of pathogens and debris in the inflamed airways.

## Abbreviations

AKT	Protein kinase B
cAMP	Cyclic adenosine monophosphate
CFTR	Cystic fibrosis transmembrane conductance regulator
COPD	Chronic obstructive pulmonary disease
Dexa	Dexamethasone
GCs	Glucocorticoids
GR	Glucocorticoid receptor
I_SC_	Short-circuit current
MCC	Mucociliary clearance
NDRG1	N-myc downregulated gene 1
NEDD4L	Neural precursor cell expressed, developmentally downregulated 4-like
mTORC2	Mammalian target of rapamycin complex 2
PDK1	Phosphoinositide-dependent kinase
PI3K	Phosphoinositide 3-kinase
R_te_	Transepithelial resistance
SGK1	Serum and glucocorticoid dependent kinase 1
V_te_	Transepithelial voltage
